# Clinical performance of low-shrinkage giomer compared to nanohybrid resin composite in proximal restorations after one year: a randomized clinical trial

**DOI:** 10.1038/s41405-026-00423-2

**Published:** 2026-04-13

**Authors:** Marwa I. AbdelHafez, Omar Shaalan, Heba Hamza

**Affiliations:** 1 Department of Conservative Dentistry, Faculty of Dentistry, Cairo University, Egypt; 2 Division of Conservative Dentistry, School of Dentistry, Newgiza University, Egypt

**Keywords:** Bonded restorations, Composite resin

## Abstract

**Objectives:**

The present study assesses the clinical performance and occlusal wear of low-shrinkage giomer and nanohybrid composite in proximal restorations.

**Methods:**

Fifty participants with proximal caries in posterior teeth (*n* = 25) were recruited. Teeth were restored with either conventional nano-hybrid composite (Filtek Z250XT, 3M ESPE, USA) or low-shrinkage giomer (Beautifil II LS, Shofu Inc, Japan). Clinical performance was assessed using revised FDI criteria at baseline, 6 and 12 months. Wear was assessed after 12 months by using 3D inspection and metrology software (Geomagic Control X; 3D Systems, USA). FDI scores were analyzed using Chi‑square test for intergroup comparisons and Cochran’s Q‑test for intragroup comparisons. Wear data were analyzed using independent *t* test for intergroup comparison and paired *t* test for intragroup comparisons.

**Results:**

At the 12-month follow-up, both Beautifil II LS and Filtek Z250XT exhibited high clinical success rates, with 100% and 96% success rates, respectively. Beautifil II LS restorations had a 4% lower risk of failure compared to Filtek Z250XT (ARR = 4.0 (95% CI −12.6 to 19.5, *P* > 0.05)). The mean wear after 12 months was 0.036 ± 0.01 mm for Beautifil II LS and 0.038 ± 0.01 mm for Filtek Z250XT. The difference between groups was minimal (0.0026 mm; 95% CI: –0.0271 to 0.0324) and not statistically significant (*P* = 0.8178).

**Conclusion:**

Low-shrinkage giomer showed satisfactory clinical performance and wear resistance compared to nanohybrid resin composite after one year. Both materials are considered clinically acceptable as per the American Dental Association (ADA) standards.

## Introduction

Over the past decade, the state of art of composite materials has undergone substantial advancements driven by superior esthetic outcomes and ease of handling. Major developments in composite restorations have predominantly focused on modifications to the monomer composition, filler technology, and initiator systems [1]. Filler type, for instance, plays a critical role in determining radiopacity and mechanical performance, while also contributing to improved translucency and handling characteristics, including consistency, polishability, and long-term gloss stability [2]. Additionally, continuous reduction in filler particle size has been pursued to enhance initial surface polish while maintaining gloss over time. These innovations have led to the progressive evolution of composites from hybrid, microhybrid, and microfilled formulations till reaching nano-sized composites [3].

Despite the merits of nanohybrid composites, resin composite material still faces certain challenges, such as technique sensitivity, lack of antibacterial property and polymerization shrinkage [4]. Polymerization shrinkage is perhaps one of the most critical problems of direct restorative materials. Polymerization shrinkage of resin-based composites ranges from 2.6 to 7.1% [5]. Polymerization stresses can lead to microleakage, marginal discoloration, postoperative sensitivity, stress at the tooth-restoration interface, and secondary caries [6]. Moreover, the absence of antibacterial property and remineralization potential are another critical factors that may increase the possible incidence of secondary caries around restorations [4].

In the early 2000s, Shofu (Kyoto, Japan) developed a line of fluoride-releasing materials called “giomer”. Giomers are considered true hybridization of glass ionomer and composite resin, containing surface pre-reacted glass ionomer (S-PRG) filler particles within a resin matrix [7]. These S-PRG fillers allows the material to release fluoride and be recharged with fluoride, which in theory makes it able to release fluoride over long term [8]. Moreover, this technology enables the controlled release of multiple therapeutic ions other than fluoride, including strontium, sodium, borate, aluminum, and silicate contributing to remineralization, acid buffering, antimicrobial activity, and inhibition of demineralization [9].

Furthermore, the continuous modifications and changes in composite formulations have resulted in expanded range of giomer materials including Beautifil II, a bioactive restorative composite, with improved filler loading, allowing for enhanced polishability and shade stability [7]. Recently, low-shrinkage giomer resin composite (LS-GRC) was introduced to the market enhanced by their low-shrinkage property through implementing filler technology in the matrix system. According to the manufacturer, low-shrinkage giomer resin composite (LS-GRC) demonstrates volumetric shrinkage of 0.8% and polymerization shrinkage stress of 2.72 MPa, which contributes to superior clinical performance over time [10].

Several studies have been conducted to evaluate the overall clinical performance of LS-GRC with comparable success rates when compared to other restorative materials [11, 12]. However, further research is still needed to evaluate the performance and quantitative amount of wear after placement of LS-GRC in stress-bearing areas. Assessing wear behavior of composite materials is crucial as excessive occlusal wear may result in loss of occlusal morphology, marginal breakdown, loss of vertical dimension, food impaction, and deterioration of functional and esthetic performance of the restoration [13]. Traditional wear assessment methods, such as study casts, clinical examination, and intraoral photographs, have limited accuracy in detecting early or minor wear changes. In contrast, digital intraoral scanning enables precise three-dimensional quantitative analysis of wear allowing for reliable longitudinal comparisons [14].

Therefore, the aim of the present study was to assess the clinical performance and wear behavior of LS-GRC versus nanohybrid composite in proximal restorations. The null hypothesis tested was that there would be no difference between the two tested materials after one year in proximal restorations of posterior teeth.

## Materials and methods

### Study settings, trial registration, ethical approval and trial design

The current randomized clinical trial was held in the Faculty of Dentistry, Cairo University, Egypt. All procedures performed in this study, involving human participants, were in accordance with the principles of the Declaration of Helsinki in 2013 and the ethical standards of Research Ethics Committee of Faculty of Dentistry, Cairo University (REC), (Approval no. 8-7-23). The present clinical trial was registered in (www.clinicaltrials.gov) under registration I.D (NCT05949502) at 24-06-2023. The trial design was double-blind, randomized clinical trial (RCT) in parallel arm design. The trial framework was superiority frame with an allocation ratio of 1:1. The present trial was reported according to CONSORT (Consolidated Standards of Reporting Trials) 2025 guidelines [15].

### Sample size collection

The sample size was calculated based on a previous study [16] in which success rate of nanohybrid composites in proximal posterior restorations was 100%. A two-tailed Z test for the difference between two independent proportions was applied, with an alpha level of 5% and a power of 80%. In order to detect a difference of 30%, the required sample size was calculated as 22 per group. To compensate for possible dropouts, sample size was raised by 15% to reach 25, with a total of 50 participants. The sample size calculation was performed using G*Power software (version 3.1.9.2 for Windows).

### Eligibility criteria

Participants ranging from 19 to 30 years, with good oral hygiene, stable occlusion, healthy periodontium, compliance and could be present for further periodic follow-ups were included. Vital first and second molars or premolars with moderately sized compound class II lesions involving 2/3 of the dentin thickness (Si/Sta 2.2) were included. The selected teeth were in occlusal contact with natural dentition and exhibiting proximal contact with the adjacent teeth.

On the other hand, patients with poor oral hygiene, heavy occlusal stresses, heavy smokers, patients with xerostomia, or participating in another clinical trial were excluded from the study. The exclusion criteria of teeth were fracture, evidence of crack, periapical pathosis and teeth adjacent or opposing to defective restorations.

### Recruitment

Participants were recruited from the diagnostic center, Faculty of Dentistry, Cairo University between 15/07/2023 and 30/09/2023 according to the eligibility criteria using convenient consecutive sampling till the required sample size was fulfilled. All participants in the trial approved and signed the written informed consent after acceptance to participate.

### Randomization, allocation concealment, and blinding

Allocation sequence was determined using simple randomization through generating random allocation sequence using (https://www.random.org). Sequence was created by generating random numbers from 1 to 50 into two columns. Participants were randomly allocated according to the assigned group. Each participant selected a random number from a sealed, opaque envelope [17]. Participants and outcome assessors were blinded to the materials’ assignment, while difference in the application protocol prohibited blinding of the operator.

### Participants’ preparation

Before any restorative procedures, the selected teeth were polished, followed by preoperative occlusal assessment, periapical and bitewing radiographs, and vitality testing using a pulp vitality tester (Parkell Pulp Vitality Tester, Parkell Electronics DN, Farmingdale, NY, USA).

### Field isolation and cavity preparation procedures

Local anesthesia was administrated prior to cavity preparation using Artpharmadent 1:100,000 (Artpharma, Egypt). All restorative procedures were done in a multiple isolation technique using rubber dam (Sanctuary Dental Dam, Sanctuary Health Sdn Bhd, Perak, Malaysia) with suitable clamps (KSK Clamps, Dentech Corporation, Tokyo, Japan) [18]. Cavities were prepared using sterile #330 and #245 carbide burs (Mani Inc., Tochigi, Japan) running at high speed (380,000–450,000 rpm) under profuse coolant, burs were discarded after five cavities [19]. Caries was removed according to the International Caries Consensus Collaboration (ICCC) [20]. Soft caries was excavated by a small, sharp excavator (Dentsply® Maillefer, Ballaigues, Switzerland) till reaching a firm dentin. Enamel walls were finished after cavity preparation using high-speed, yellow-coded diamond stone (Dia-burs, Mani Inc., Tochigi, Japan).

A sectional matrix system (Composi-Tight 3D Fusion Sectional Matrix System, Garrison Dental Solutions, Spring Lake, Michigan, USA) was used to restore the proximal wall. The sectional matrix size was chosen according to the size of the prepared cavity, then a wedge was chosen and placed according to the size of the cervical embrasure. Further, a suitable-sized ring was placed over them using the ring placement forceps [21].

### Interventions

All materials used in the study were placed in accordance with the manufacturers’ instructions (Table 1). Selective etching technique (Scotchbond Universal Etchant, 3M ESPE, Germany) was applied using a 35% phosphoric acid gel on the enamel margins surrounding the whole cavity for 15 s followed by rinsing for 15 s and air drying using a water/oil-free air for 5 s. The excess moisture was blot dried using a moist cotton pellet [22].

**Table 1:** Brand, type, chemical composition and lot no. of used materials.

Brand	Type	Chemical composition	Lot number
Scotchbond Universal Etchant (3M Oral Care)	Etching gel	Phosphoric acid 35%, water, synthetic amorphous silica	466293
Beautifil II LS (Shofu Inc, Kyoto, Japan)	Low-shrinkage Giomer resin composite	Multifunctional glass and S-PRG filler based on fluoro-boro-aluminosilicate glass, pre-polymerized filler, nano filler, photo-initiator, low-shrinkage urethane diacrylate, bis-MPEPP, bis-GMA, TEGDMA	062269
BeautiBond Xtreme (Shofu Inc, Kyoto, Japan)	Universal adhesive	Bisphenol-A-diglycidyl methacrylate (10–20%), Triethylene-glycol dimethacrylate (<10%), Acid monomer (<20%), Acetone and water (65–85%), Silane coupling agent (<5%), Others (<5%)	072355
Filtek Z250XT (3 M ESPE, St. Paul, MN, USA)	Nano-hybrid composite	Organic matrix: bisphenol A-glycidyl methacrylate, urethane dimethacrylate, bisphenol A polyethylene glycol diether-dimethacrylate, triethylene-glycol dimethacrylate Inorganic filler: zirconia/silica, Aluminum oxide	9712886
Single bond universal (3M ESPE, Neuss, Germany)	Universal adhesive	10-MDP phosphate monomer, Vitrebond, copolymer, HEMA, Bis-GMA, dimethacrylate resin, silane, ethanol, water.	30704B

### Low-shrinkage giomer

Universal adhesive (BeautiBond Xtreme, Shofu, Japan) was applied to the cavity walls for 10 s, gently air-blown with water/oil-free air for 3 s until the surface appeared glossy, then photopolymerized for 10 s using LED light curing unit (LED F, Woodpecker Medical Instrument Co., Ltd, Guangdong, China) with an output of 1600–1800 mW/cm^2^ [23]. The output was checked regularly using radiometer between participants. Flowable composite (Beautifil Flow Plus X F03, Shofu Inc, Kyoto, Japan) was applied on the gingival seat as a first increment followed by low-shrinkage bioactive giomer material (Beautifil II LS, Shofu Inc, Kyoto, Japan) by snow-plow technique [24]. Centripetal technique was performed to restore the proximal wall followed by oblique incrementation of ~2 mm thick composite resin on each cusp using a gold-plated applicator (Nordent Manufacturing Inc. Illinois, USA). This was followed by light curing for 20 s of each increment till the whole cavity is filled [25].

### Nanohybrid composite

A single layer from the adhesive (Single Bond Universal, 3M ESPE, Germany) was actively agitated for 20 s on the entire cavity, then gently air-blown with water/oil-free air for 5 s till reaching a glossy surface that cannot be visibly moved under further air pressure [26]. The adhesive was light-cured for 10 s using LED light-curing unit. Nano-hybrid flowable composite (Filtek™ Supreme XTE Flow, 3M ESPE, St. Paul, MN, USA) was applied on the gingival seat as a first increment followed by application of nano-hybrid universal composite (Filtek Z250XT, 3M ESPE, St. Paul, MN, USA) by snow-plow technique [24]. The proximal wall was restored first by centripetal technique followed by oblique incrementation of ~2 mm thick composite resin according to the manufacturer instructions [25]. Light curing was performed for 20 s on each cusp till the whole cavity was filled.

### Finishing and polishing

Restorations were finished by a yellow-coded flame stone (Dia-burs, Mani Inc., Tochigi, Japan) rotating at high-speed under water coolant. Occlusion was checked and adjusted using a double sided, 35 microns, articulating paper (Accufilm II, Parkell Inc., New York, USA). Fine and super-fine diamond points were used for removal of excess composite flashes. Polishing was performed using pre-impregnated rubber cups under intermittent water spray (OneGloss PS, Shofu Dental Corportation, California, USA) [27, 28].

### Outcome assessment

#### Clinical assessment

The primary outcome of this study was clinical performance of the restorations using revised FDI criteria, while the secondary outcome was measuring the restoration wear quantitatively using digital intra-oral scanner and 3D inspection software (Geomagic Control X, 3D Systems, Rock Hill, SC, USA) [14, 29].

The revised FDI criteria was used to evaluate the quality of the restorations over time through a set of standardized criteria covering functional parameters (fracture and retention (F1), marginal adaptation (F2), proximal contact (F3), form and contour (F4), occlusion and wear (F5)); biological parameters (caries at the restoration margin (B1), dental hard tissue defects at restoration margin (B2), and postoperative hypersensitivity (B3)), and esthetic parameters (surface luster and texture (A1), marginal staining (A2), and color matching (A3)). Restorations were rated on a five-point scale ranging from excellent (1) to poor (5). Scores between 1 and 3 were considered clinically acceptable, a score of 4 indicated an unacceptable restoration that could be repaired, and a score of 5 denoted an unacceptable restoration where repair was not feasible [29].

**Fig. 1:**
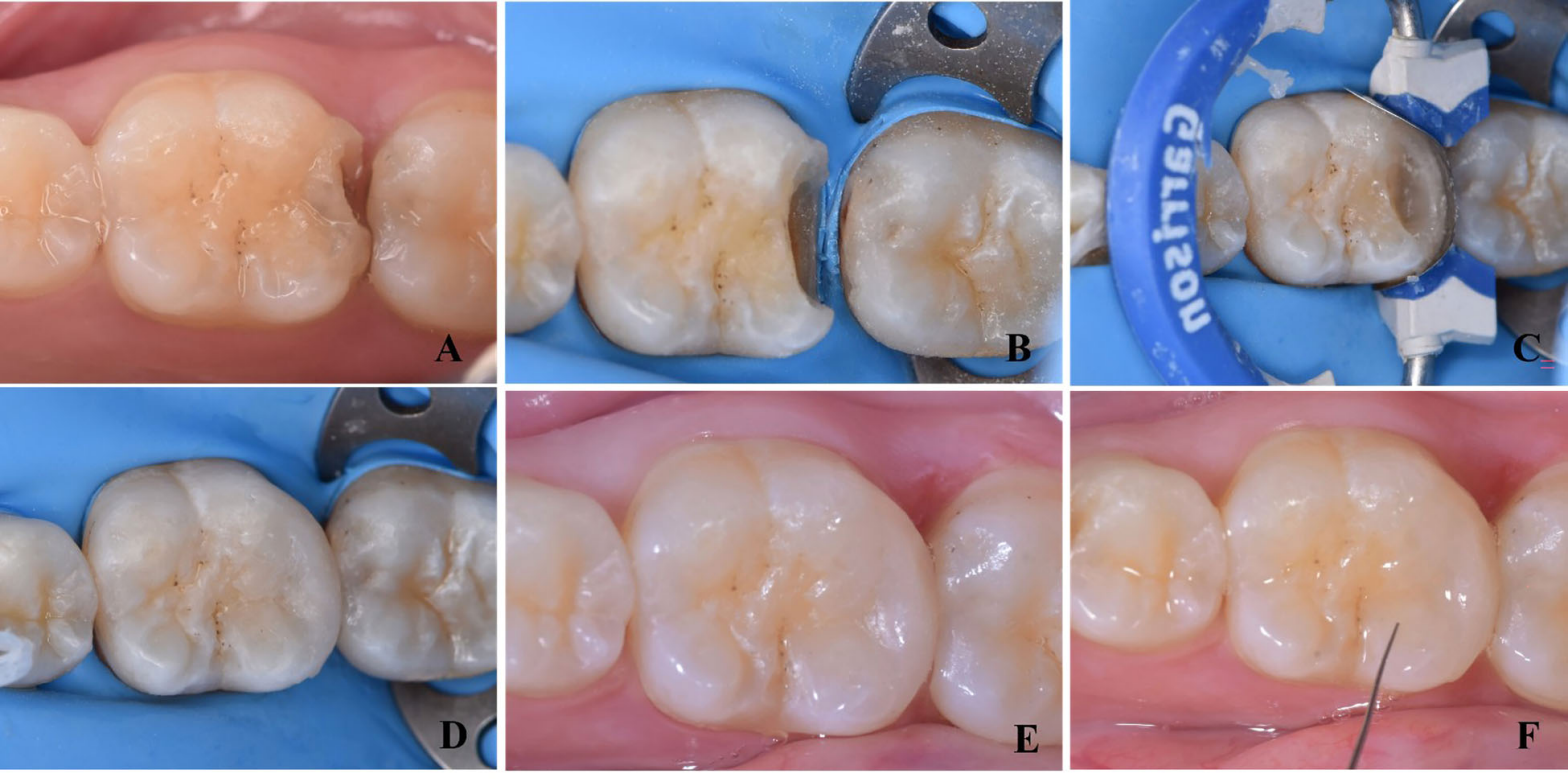
Representative clinical photos (Beautifil II LS group). **A** Pre-operative view of tooth 46, **B** after cavity preparation, **C** After matrix placement and building the proximal wall, **D** Immediately after placement of low-shrinkage Giomer resin composite, **E** Follow-up periods after 6 months, **F** After 12 months.

Each restoration was assessed by two experienced blinded assessors with more than 15 years of expertise in restorative dentistry. The evaluators were trained and calibrated prior to evaluation on using the revised FDI criteria for assessment of clinical performance. Assessments were performed at baseline, 6- and 12-month follow-up. Any discrepancies between examiners were resolved through discussion to reach a consensus. Figure 1 shows a representative case using LS-GRC for all procedural steps and follow-up assessments.

### Wear assessment (quantitative analysis)

**Fig. 2:**
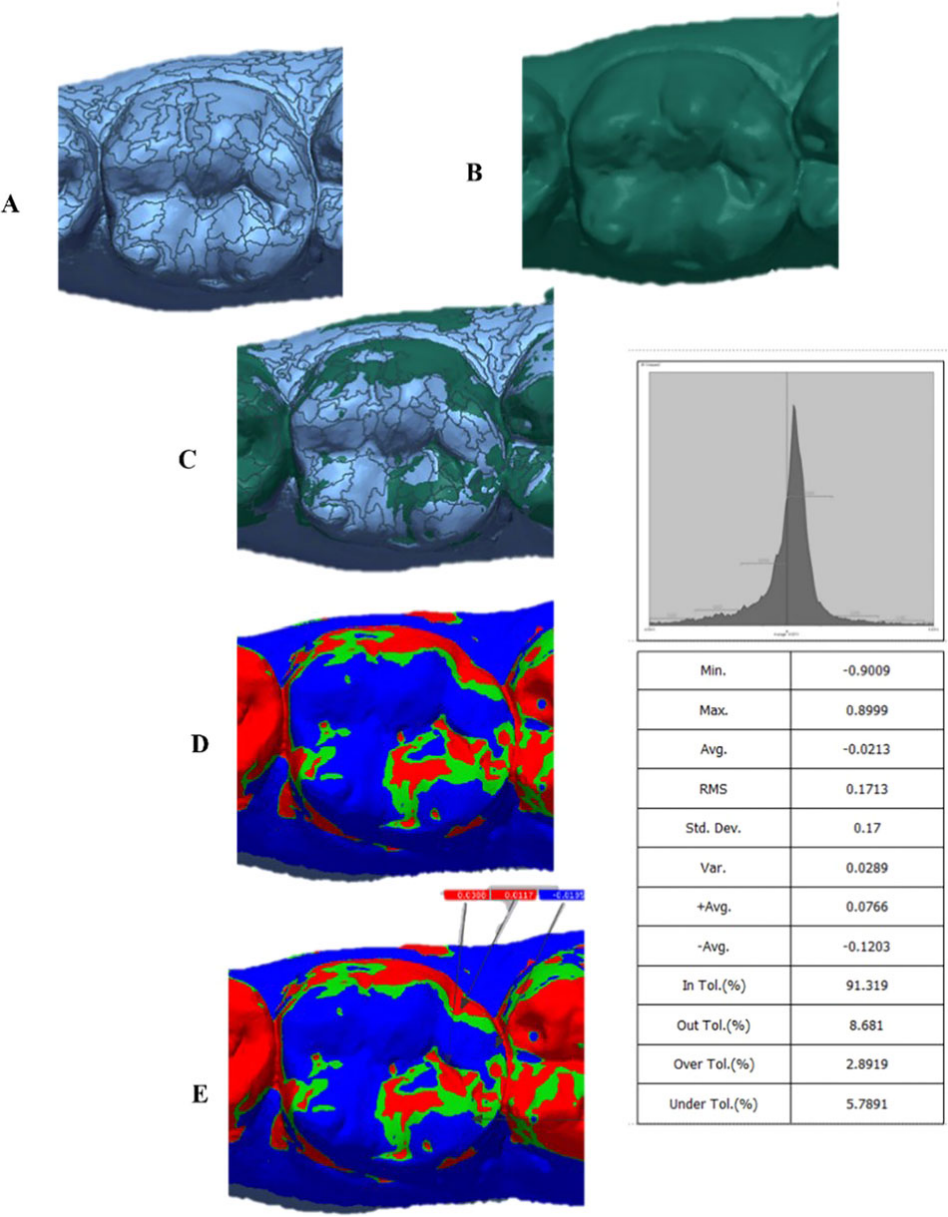
Schematic presentation showing steps of measuring the amount of occlusal wear after one year follow-up using Geomagic Control X software. **A** Baseline intra-oral scan; **B** Intra-oral scan after one year; **C** Combined STL files using best-fit alignment method; **D** 3D compare method between the two scans; **E** Comparison at different points between the baseline and after one year.

For each case, baseline records and records after 1 year were obtained using CEREC Omnicam® digital intraoral scanner (Dentsply Sirona, York, Pennsylvania, USA). Data were exported into standard tessellation language (STL) format, then introduced to Geomagic Control X software (3D Systems, Rock Hill, SC, USA). The two scans were superimposed using “initial alignment” then “best-fit alignment” process. Root mean square (RMS) values were then calculated using “3D-compare” method, this was followed by comparison at different surface points (buccal, palatal, central, marginal ridge) between the reference baseline record and after one year providing a colored map with a deviation of ±0.01 mm. The green areas denoted superior matching, while the red to yellow areas indicated positive positioning of the follow-up to the baseline, light to dark blue areas donated negative positioning of the follow-up to the baseline [30]. The root mean square (RMS) values were calculated as ${RMS}=\frac{1}{\sqrt{n}}\sqrt{{\sum }_{i=1}^{n}\left({x}_{1},i-{x}_{2},i\right)\,2.}$ [14, 31] (Fig. 2).

### Statistical analysis

Data analysis was conducted using MedCalc software version 22 (MedCalc Software Ltd, Ostend, Belgium). Categorical data were presented as frequencies and percentages. Intergroup comparisons were carried out using Chi-squared test (*P* ≤ 0.05), while intragroup comparisons within each intervention were performed using Cochran’s Q test with Bonferroni correction (*P* ≤ 0.016). Clinical effect size was assessed by calculating absolute risk reduction. Survival analysis was performed using Kaplan–Meier analysis followed by log-rank test. Wear data were analyzed using an independent *t*-test for intergroup comparison and paired *t*-test for intragroup comparisons (*P* ≤ 0.05).

### Ethics approval and consent to participate

The study protocol was registered at clinicaltrials.gov under the identifier NCT05949502 (24-06-2023). Ethical approval was obtained from the Research Ethics Committee of the Faculty of Dentistry, Cairo University (approval ID: 8-7-23), in accordance with the principles outlined in the Declaration of Helsinki (2013).

## Results

### Demographic data

In the current study, 50 participants with proximal carious lesions were randomly assigned to the intervention and control arms (*n* = 25). After 12 months, all participants were assessed with 100% retention rate. The mean age of participants was 30.6 ± 5.56 years; 30.0 ± 5.02 years in the Beautifil II LS group and 31.2 ± 6.10 years in the Filtek Z250XT group. Gender distribution was comparable, with Beautifil II LS having 8 males (32%) and 17 females (68%), and Filtek Z250XT having 11 males (44%) and 14 females (56%).

**Fig. 3:**
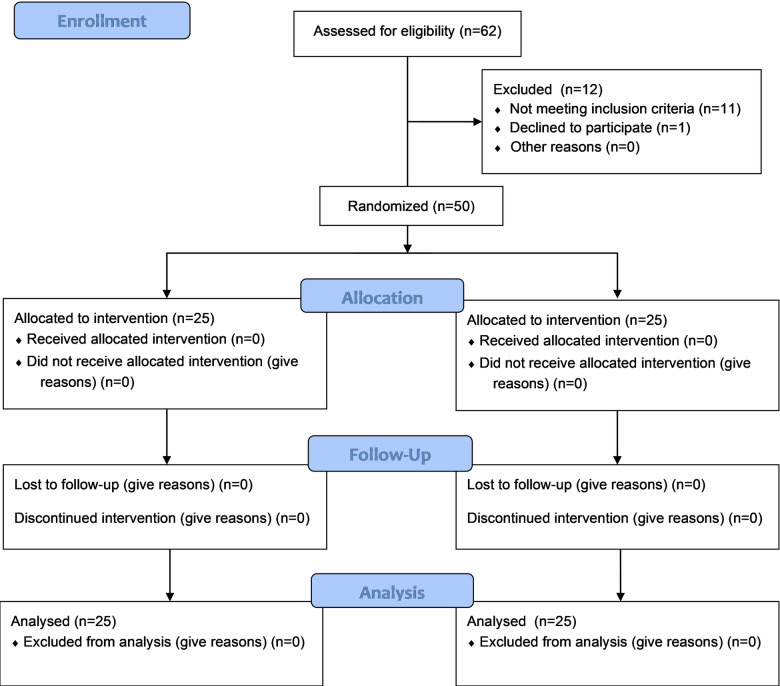
CONSORT 2025 flow diagram.

Regarding tooth type, Beautifil II LS restorations were placed in maxillary premolars (16%), maxillary molars (16%), mandibular premolars (36%), and mandibular molars (32%), while Filtek Z250XT restorations were placed in maxillary premolars (36%), maxillary molars (20%), mandibular premolars (8%), and mandibular molars (36%). There were no statistically significant differences between both groups for age, gender, or tooth distribution (*P* > 0.05) (Table 2). Figure 3 shows the Consort 2025 flow diagram of participants and teeth in the current trial [15].

**Table 2:** Demographic data.

	Beautifil LS (*n* = 25)	Filtek Z250XT (*n* = 25)	*P* value
Age, mean ± SD	30.0 ± 5.02	31.2 ± 6.10	0.4516
Gender, *n*(%)			0.3869
Male	8 (32)	11 (44)	
Female	17 (68)	14 (56)	
Teeth, *n*(%)			0.0878
Maxillary premolar	4 (16)	9 (36)	
Maxillary molar	4 (16)	5 (20)	
Mandibular premolar	9 (36)	2 (8)	
Mandibular molar	8 (32)	9 (36)	

### Assessment of clinical performance

**Fig. 4:**
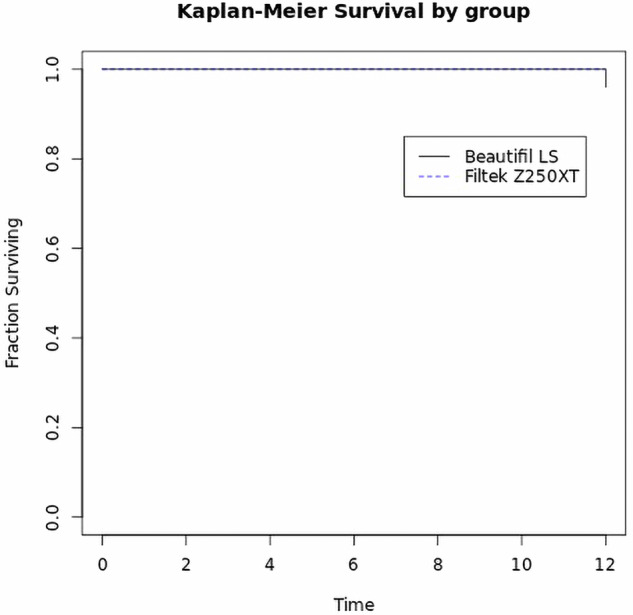
Survival analysis of both materials in proximal restorations at 12 months.

**Fig. 5:**
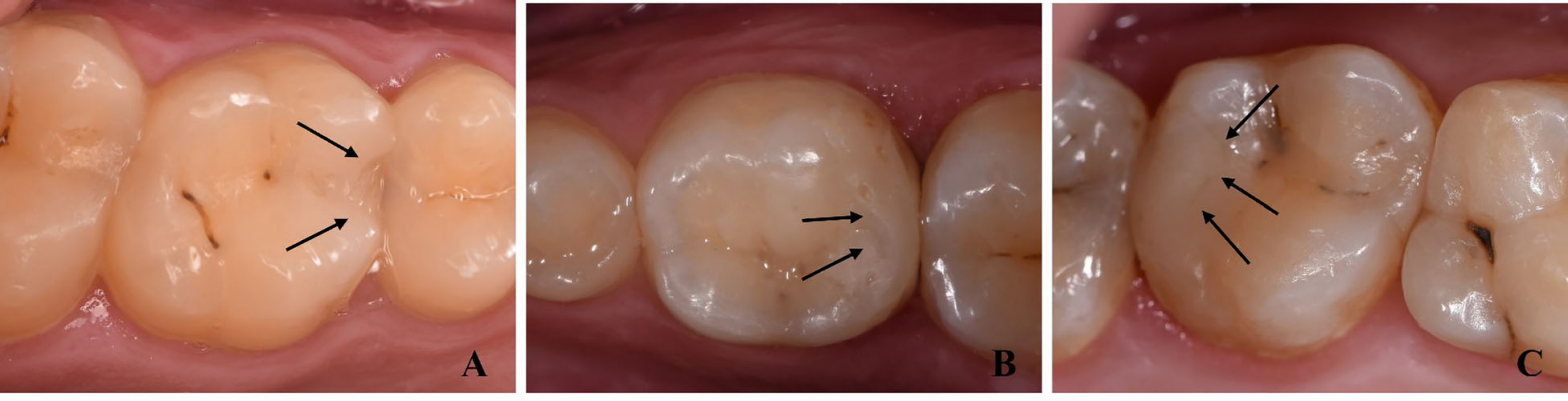
Minor deviations from ideal scores at 12-month follow-up period. **A** Material chipping (F1) score 2 (Beautifil II LS group), **B** Slight loss of surface luster (A1) score 2 (Filtek Z250XT group), **C** Minor marginal staining detectable after air drying (A2) score 2 (Filtek Z250XT group).

After 12 months, both Beautifil II LS and Filtek Z250XT exhibited high clinical success rates. Beautifil II LS group showed 100% success rate, whereas one restoration in the Filtek Z250XT group failed, scoring 5 in fracture of material and retention (F2), resulting in a 96% success rate, Log-rank test showed no statistically significant difference between both materials (*P* = 0.3173) (Fig. 4). Beautifil II LS restorations had a 4% lower risk of failure compared to Filtek Z250XT (ARR = 4.0 (95% CI −12.6 to 19.5, *P* > 0.05)). Minor deviations from ideal scores were observed in the Beautifil II LS group, where four restorations scored 2; two for postoperative hypersensitivity (B3) and two for fracture and retention (F1). In the Filtek Z250XT group, six restorations scored 2 across multiple criteria, including surface luster (A1), marginal staining (A2), color match (A3), marginal adaptation (F2), proximal contact (F3), form and contour (F4), and occlusion/wear (F5) (Fig. 5). Overall, both materials demonstrated excellent clinical performance over the 12-month period, with no significant difference for all assessed criteria (*P* > 0.05) (Table 3).

**Table 3:** Clinical performance according to revised FDI criteria

FDI	Follow-up	Beautifil LS					Filtek Z250XT					*P* value
		Success			Failure		Success			Failure		
Score		1	2	3	4	5	1	2	3	4	5	
A1	Baseline	25 (100.0)	0 (0.0)	0 (0.0)	0 (0.0)	0 (0.0)	25 (100.0)	0 (0.0)	0 (0.0)	0 (0.0)	0 (0.0)	1.0000
	6 months	25 (100.0)	0 (0.0)	0 (0.0)	0 (0.0)	0 (0.0)	24 (96.0)	1 (4.0)	0 (0.0)	0 (0.0)	0 (0.0)	0.3173
	12 months	25 (100.0)	0 (0.0)	0 (0.0)	0 (0.0)	0 (0.0)	22 (91.7)	2 (8.3)	0 (0.0)	0 (0.0)	0 (0.0)	0.1447
	*P* value	1.0000					0.368					
A2	Baseline	25 (100.0)	0 (0.0)	0 (0.0)	0 (0.0)	0 (0.0)	25 (100.0)	0 (0.0)	0 (0.0)	0 (0.0)	0 (0.0)	1.0000
	6 months	25 (100.0)	0 (0.0)	0 (0.0)	0 (0.0)	0 (0.0)	24 (96.0)	1 (4.0)	0 (0.0)	0 (0.0)	0 (0.0)	0.3173
	12 months	25 (100.0)	0 (0.0)	0 (0.0)	0 (0.0)	0 (0.0)	22 (91.7)	2 (8.3)	0 (0.0)	0 (0.0)	0 (0.0)	0.1447
	*P* value	1.0000					0.368					
A3	Baseline	25 (100.0)	0 (0.0)	0 (0.0)	0 (0.0)	0 (0.0)	25 (100.0)	0 (0.0)	0 (0.0)	0 (0.0)	0 (0.0)	1.0000
	6 months	25 (100.0)	0 (0.0)	0 (0.0)	0 (0.0)	0 (0.0)	23 (92.0)	2 (8.0)	0 (0.0)	0 (0.0)	0 (0.0)	0.1530
	12 months	25 (100.0)	0 (0.0)	0 (0.0)	0 (0.0)	0 (0.0)	23 (95.8)	1 (4.2)	0 (0.0)	0 (0.0)	0 (0.0)	0.3074
	*P* value	1.0000					0.368					
B1	Baseline	25 (100.0)	0 (0.0)	0 (0.0)	0 (0.0)	0 (0.0)	25 (100.0)	0 (0.0)	0 (0.0)	0 (0.0)	0 (0.0)	1.0000
	6 months	25 (100.0)	0 (0.0)	0 (0.0)	0 (0.0)	0 (0.0)	25 (100.0)	0 (0.0)	0 (0.0)	0 (0.0)	0 (0.0)	1.0000
	12 months	25 (100.0)	0 (0.0)	0 (0.0)	0 (0.0)	0 (0.0)	24 (100.0)	0 (0.0)	0 (0.0)	0 (0.0)	0 (0.0)	0.8864
	*P* value	1.0000					0.368					
B2	Baseline	25 (100.0)	0 (0.0)	0 (0.0)	0 (0.0)	0 (0.0)	25 (100.0)	0 (0.0)	0 (0.0)	0 (0.0)	0 (0.0)	1.0000
	6 months	25 (100.0)	0 (0.0)	0 (0.0)	0 (0.0)	0 (0.0)	25 (100.0)	0 (0.0)	0 (0.0)	0 (0.0)	0 (0.0)	1.0000
	12 months	25 (100.0)	0 (0.0)	0 (0.0)	0 (0.0)	0 (0.0)	24 (100.0)	0 (0.0)	0 (0.0)	0 (0.0)	0 (0.0)	0.8864
	*P* value	1.0000					0.368					
B3	Baseline	25 (100.0)	0 (0.0)	0 (0.0)	0 (0.0)	0 (0.0)	25 (100.0)	0 (0.0)	0 (0.0)	0 (0.0)	0 (0.0)	1.0000
	6 months	23 (92.0)	2 (8.0)	0 (0.0)	0 (0.0)	0 (0.0)	24 (96.0)	1 (4.0)	0 (0.0)	0 (0.0)	0 (0.0)	0.5555
	12 months	23 (92.0)	2 (8.0)	0 (0.0)	0 (0.0)	0 (0.0)	24 (100.0)	0 (0.0)	0 (0.0)	0 (0.0)	0 (0.0)	0.1614
	*P* value	1.0000					0.368					
F1	Baseline	25 (100.0)	0 (0.0)	0 (0.0)	0 (0.0)	0 (0.0)	25 (100.0)	0 (0.0)	0 (0.0)	0 (0.0)	0 (0.0)	1.0000
	6 months	25 (100.0)	0 (0.0)	0 (0.0)	0 (0.0)	0 (0.0)	24 (96.0)	1 (4.0)	0 (0.0)	0 (0.0)	0 (0.0)	0.3173
	12 months	23 (92.0)	2 (8.0)	0 (0.0)	0 (0.0)	0 (0.0)	24 (96.0)	0 (0.0)	0 (0.0)	0 (0.0)	1 (4.0)	0.2208
	*P* value	1.0000					0.368					
F2	Baseline	25 (100.0)	0 (0.0)	0 (0.0)	0 (0.0)	0 (0.0)	25 (100.0)	0 (0.0)	0 (0.0)	0 (0.0)	0 (0.0)	1.0000
	6 months	25 (100.0)	0 (0.0)	0 (0.0)	0 (0.0)	0 (0.0)	24 (96.0)	1 (4.0)	0 (0.0)	0 (0.0)	0 (0.0)	0.3173
	12 months	25 (100.0)	0 (0.0)	0 (0.0)	0 (0.0)	0 (0.0)	23 (95.8)	1 (4.2)	0 (0.0)	0 (0.0)	0 (0.0)	0.3074
	*P* value	1.0000					0.368					
F3	Baseline	25 (100.0)	0 (0.0)	0 (0.0)	0 (0.0)	0 (0.0)	25 (100.0)	0 (0.0)	0 (0.0)	0 (0.0)	0 (0.0)	1.0000
	6 months	25 (100.0)	0 (0.0)	0 (0.0)	0 (0.0)	0 (0.0)	24 (96.0)	1 (4.0)	0 (0.0)	0 (0.0)	0 (0.0)	0.3173
	12 months	25 (100.0)	0 (0.0)	0 (0.0)	0 (0.0)	0 (0.0)	23 (95.8)	1 (4.2)	0 (0.0)	0 (0.0)	0 (0.0)	0.3074
	*P* value	1.0000					0.368					
F4	Baseline	25 (100.0)	0 (0.0)	0 (0.0)	0 (0.0)	0 (0.0)	25 (100.0)	0 (0.0)	0 (0.0)	0 (0.0)	0 (0.0)	1.0000
	6 months	25 (100.0)	0 (0.0)	0 (0.0)	0 (0.0)	0 (0.0)	25 (100.0)	0 (0.0)	0 (0.0)	0 (0.0)	0 (0.0)	1.0000
	12 months	25 (100.0)	0 (0.0)	0 (0.0)	0 (0.0)	0 (0.0)	23 (95.8)	1 (4.2)	0 (0.0)	0 (0.0)	0 (0.0)	0.3074
	*P* value	1.0000					0.368					
F5	Baseline	25 (100.0)	0 (0.0)	0 (0.0)	0 (0.0)	0 (0.0)	25 (100.0)	0 (0.0)	0 (0.0)	0 (0.0)	0 (0.0)	1.0000
	6 months	25 (100.0)	0 (0.0)	0 (0.0)	0 (0.0)	0 (0.0)	24 (96.0)	1 (4.0)	0 (0.0)	0 (0.0)	0 (0.0)	0.3173
	12 months	25 (100.0)	0 (0.0)	0 (0.0)	0 (0.0)	0 (0.0)	23 (95.8)	1 (4.2)	0 (0.0)	0 (0.0)	0 (0.0)	0.3074
	*P* value	1.0000					0.368					

### Assessment of wear

The mean wear after 12 months was 0.036 ± 0.01 mm for Beautifil II LS and 0.038 ± 0.01 mm for Filtek Z250XT. The difference between groups was minimal (0.0026 mm; 95% CI: –0.0271 to 0.0324) and not statistically significant (*P* = 0.8178).

## Discussion

The continuous modifications and changes in composite formulations by dental manufacturers have resulted in an expanded range of restorative materials with favorable long-term clinical performance [32]. The clinical use of fluoride-releasing adhesive restorative materials has risen significantly. These materials include giomer, which is valued for successfully integrating the protective advantages of conventional glass ionomers with the desirable characteristics of resin composites [8]. Giomer materials utilize S-PRG (Surface Pre-Reacted Glass ionomer) fillers that are notable for releasing multiple types of ions, such as F^-^, Al^3+^, BO_3_^3-^, Na^+^, SiO_3_^2^, and Sr^2+^ ions which are beneficial for remineralization and by acting as a pH modulator; they help to neutralize the acidity in the surrounding oral environment following an acid attack [7]. Previous studies have evaluated the clinical performance of giomer compared to other restorative materials [11, 33, 34], yet no studies evaluated the clinical performance alongside assessment of quantitative amount of wear.

Wear resistance is an essential property of dental restorative materials, as restorations should ideally exhibit wear behavior comparable to natural teeth to maintain long-term occlusal stability [13]. Although natural teeth show relatively low annual occlusal wear, resin composites demonstrate higher wear rates, particularly during the first five years after placement, which may be related to the composite material itself, or patient-related factors and evaluation methods [35]. Quantitative assessment of occlusal wear is considered a sensitive method for evaluation of wear resistance of composite restorations [14]. Digital scanning followed by analysis software has been widely employed in previous studies effectively for recording the amount of occlusal wear objectively, therefore eliminating issues with physical storage of measurement indices [36].

According to the manufacturer instructions, adhesives and restorative materials from the same manufacturer were used as these systems are chemically designed and validated to function synergistically as a restorative system, thereby producing more stable bond strength by minimizing the risk of cross-incompatibility [37]. In the present study, both systems were applied in selective enamel etching technique to ensure optimal adhesion between tooth structure and the resin composite [22]. The presence of functional monomer (10-MDP phosphate monomer) in the single bond universal offers stable calcium salts with hydroxyapatite contributing to a more durable chemical bond with the tooth structure [26]. While carboxylic and phosphonic acid monomers in BeautiBond Xtreme promote strong chemical bond with tooth structure, enhancing resistance to hydrolytic degradation and contributing to long-term stability of the adhesive interface [23].

According to the results of the current study, the two materials exhibited similar acceptable clinical outcomes when evaluated for their biological compatibility, functional performance, and aesthetic appearance. These results were in accordance with recent literature, which has shown that fluoride-releasing composites and traditional resin composites exhibit comparable mechanical, biological and esthetic properties, consequently, the null hypothesis could not be rejected [12, 38]. Regarding functional properties, four restorations in the Beautifil II LS group, scored 2 for fracture and retention (F1), while in Filtek Z250XT group, one restoration scored 5 for fracture and retention exhibiting complete loss of the restoration due to adhesive failure. This failure may be attributed to the thickness of the adhesive layer during bonding procedure. Previous literature [39] denoted that excessive thinning of the adhesive layer exceeding the thickness of the oxygen-inhibited layer may result in compromising the hybrid layer formation, thereby reducing the mechanical integrity of the bonding interface and potentially impairing its long-term stability. In addition, six restorations scored 2 for marginal adaptation (F2), proximal contact (F3), form and contour (F4), and occlusion/wear (F5). According to Toz Alkin et al. [40], the minor deviation from ideal scores over time could be related to several factors such as technique sensitivity during application of the adhesive systems, the clinical expertise of the clinicians, and adhesive system-related factors.

Regarding esthetic properties, six restorations of Filtek Z250XT group scored 2 for surface luster (A1), marginal staining (A2), and color match (A3). These results were consistent with a recent systematic review [41], who reported minor changes in surface texture and staining of some nanohybrid restorations after finishing and polishing. These changes might be attributed to the exfoliation of larger filler particles from the resin matrix during polishing. Additionally, the hydrophilic nature of the resinous material might lead to noticeable color change [42]. Another study reported that voids may be entrapped during the incremental layering technique, thereby affecting surface texture. This problem is considered crucial since rougher surfaces would cause surface staining; therefore, increasing plaque retention and bacterial adhesion [43]. However, according to FDI criteria score 2 is considered clinically good, so these six restorations were considered successful [29, 44].

Regarding biological properties, four restorations in the Beautifil II LS group scored 2 for postoperative sensitivity (B3) indicating minor postoperative pain upon chewing one week after placement of the restorations. Although LS-GRC exhibits low tendency for volumetric shrinkage, attributed to its unique Steric Repulsion Structured (SRS) molecule, which limits shrinkage to a considerable level [10], some unrelieved stresses upon polymerization might have led to this outcome, subsequently leading to gap formation at the margins and postoperative sensitivity [12]. Another assumption by previous research linked the post-operative sensitivity of resin composite restorations to the technique sensitivity associated with the adhesion procedures [39]. According to literature, significant concerns have been raised regarding the interfacial aging associated with degradation of the adhesive interface over time [45]. On the other hand, secondary caries at the restoration margins was not reported in any of the restorations of Beautifil II LS group, aligning with a previous study [46] correlating the ability of S-PRG fillers to create an acid-resistant surface layer with the reduction in plaque accumulation and bacterial adhesion on the surface of the composite restorations. Additionally, the S-PRG fillers act as rechargeable fluoride reservoirs when exposed to fluoride toothpaste or mouth rinses, leading to continuation of the fluoride re-release process [7, 47].

Regarding quantitative wear, the mean occlusal wear after 12 months was 0.036 ± 0.01 mm for Beautifil II LS and 0.038 ± 0.01 mm for Filtek Z250XT. The difference between groups was minimal and not statistically significant (*P* = 0.8178) indicating comparable wear resistance for both materials. A previous study [48] reported that the annual enamel wear rate is ranging from 0.015 mm for premolars and 0.029 mm for molars. Another study [13] reported an annual vertical enamel loss ~0.02–0.04 mm under physiological conditions, these wear rates suggested normal physiological wear of both restorative materials over one year when compared to enamel. Filtek Z250XT showed a non-significant higher wear rate compared to Beautiful II LS, this may be correlated with the deterioration detected during clinical assessment using FDI criteria [49].

According to literature, the abrasive wear of composite resin material is influenced by multiple factors, including the size, shape, concentration, orientation, and distribution of filler particles. These factors are further modulated by masticatory forces acting on the composite, rendering the wear process highly complex [50]. Further studies have found that the abrasive wear of resin composites is minimized when filler particle size and interparticle spacing are reduced; therefore, enhancing the degree of resin polymerization and increasing the filler–matrix bond strength [51]. Both restorative materials in the current study demonstrated mean particle size of 0.02 µm and 0.4 µm for Filtek Z250XT and Beautifil II LS, respectively, which may have contributed to minimal occlusal wear over time [10, 52].

Overall, both materials demonstrated excellent clinical performance over 12-month period, with no significant difference for all assessed criteria (*P* > 0.05). According to American Dental Association (ADA) standards, adhesive restorations are considered fully acceptable only when clinical failures, including loss of restorations and microleakage, remain below 10 percent after an 18-month period, therefore both restorative materials were considered clinically acceptable after 12 months [53, 54]. These findings were in agreement with earlier research [38], they evaluated giomer resin composites in various cavity types over a follow-up period of 1–13 years and reported acceptable clinical outcomes and overall comparable morphological, functional, and mechanical performance of giomers to conventional resin composites. Further studies evaluating the performance of giomer composite material in different cavities also reported satisfactory outcomes over significant follow-up periods [33, 55].

To the best of our knowledge, this is the first clinical trial to quantitatively assess the occlusal wear of low-shrinkage giomer resin composites over a 12-month period. However, it should be highlighted that the current trial did not involve large sample size, which may have constrained the external validity of the conclusions. Moreover, a one-year follow-up duration may not fully represent the long-term behavior of the restorative materials. Therefore, larger-scale trials with longer observation periods are necessary to confirm these results and evaluate the long-term performance. Nevertheless, it is advised to consider the cost effectiveness of each restorative material and its correlation with the long-term clinical success. Finally, further research comparing giomer resin composite with other restorative materials, especially in poor oral hygiene patients is also recommended.

## Conclusion

Low-shrinkage giomer showed satisfactory clinical performance and wear resistance compared to nanohybrid resin composite after one year in proximal restorations of posterior teeth. Both materials are considered clinically acceptable as per the ADA standards.

## Data Availability

The data that supports the findings of this study are available from the corresponding author upon reasonable request.
